# Semi-automated tracking of pain in critical care patients using artificial intelligence: a retrospective observational study

**DOI:** 10.1038/s41598-021-84714-8

**Published:** 2021-03-04

**Authors:** Naoya Kobayashi, Takuya Shiga, Saori Ikumi, Kazuki Watanabe, Hitoshi Murakami, Masanori Yamauchi

**Affiliations:** 1 Department of Anesthesiology and Perioperative Medicine, Tohoku University Graduate School of Medicine, 2-1 Seiryo-machi, Aoba-ku, Sendai, Miyagi 980-8575 Japan; 2 Hitachi Solutions East Japan, Ltd., Sendai, Miyagi Japan

**Keywords:** Health care, Medical research

## Abstract

Monitoring the pain intensity in critically ill patients is crucial because intense pain can cause adverse events, including poor survival rates; however, continuous pain evaluation is difficult. Vital signs have traditionally been considered ineffective in pain assessment; nevertheless, the use of machine learning may automate pain assessment using vital signs. This retrospective observational study was performed at a university hospital in Sendai, Japan. Objective pain assessments were performed in eligible patients using the Critical-Care Pain Observation Tool (CPOT). Three machine-learning methods—random forest (RF), support vector machine (SVM), and logistic regression (LR)—were employed to predict pain using parameters, such as vital signs, age group, and sedation levels. Prediction accuracy was calculated as the harmonic mean of sensitivity, specificity, and area under the receiver operating characteristic curve (AUROC). Furthermore, 117,190 CPOT assessments were performed in 11,507 eligible patients (median age: 65 years; 58.0% males). We found that pain prediction was possible with all three machine-learning methods. RF demonstrated the highest AUROC for the test data (RF: 0.853, SVM: 0.823, and LR: 0.787). With this method, pain can be objectively, continuously, and semi-automatically evaluated in critically ill patients.

## Introduction

Patients in intensive care units (ICUs) experience a high incidence of pain, which requires detailed assessments^1–3^. It has been reported that 33% of intubated patients experience pain at rest^4^, while an additional 56% experience pain during the procedure^5^. The primary source of pain in surgical patients is the surgical site, whereas that in medical patients includes the back and extremities; notably, the incidence was reported to be no different between surgical and medical patients^1^. Stress caused by pain generally has a detrimental effect on ICU patients. Increased catecholamine levels cause arteriole vasoconstriction and decrease tissue oxygen tension, thus, resulting in tissue perfusion failure^6^. Other reactions caused by pain include increased catabolism, such as lipolysis to provide protein substrates, and muscle loss^7^. Increased catabolism associated with tissue hypoxia delays wound healing and increases the risk of infections. Pain suppresses the activity of natural killer cells^8,9^ and lowers the number of cytotoxic T-cells and phagocytic neutrophils^10^. Furthermore, acute pain is a major risk factor for subsequent chronic neuropathic pain.

In the ICU, objective methods of pain assessment, such as the Critical-Care Pain Observation Tool (CPOT)^11^ and Behavioral Pain Scale^12^ are recommended when patients cannot communicate due to sedatives, intubation, or tracheostomy^13,14^. Regular use of pain assessment scales has been reported to improve the clinical outcomes in ICU patients^15–17^; however, these methods are cumbersome and do not facilitate continuous monitoring of pain. Therefore, a method for continuous and easy assessment of patients that can enable healthcare professionals to perform interventions sooner is highly necessary.

Vital signs are considered indicators of pain; however, changes in vital signs have been reported to be nonreliable indicators of the extent of pain^9,12,18–20^. Notably, the studies that concluded the latter employed time-series data of vital signs at specific points rather than using continuous data because of computational complexity. Such data may not fully reflect the information available from vital signs because they cannot account for continuous changes in the pain.

In contrast, artificial intelligence (AI)-based predictive tools are increasingly resulting in automation of diagnostics through comprehensive monitoring of patient health, which is traditionally done by physicians and nurses, based on real-time and continuous recordings, processing, monitoring, and intelligent diagnosis^21^. Notably, AI-based tools have the potential to provide continuous and automated pain assessments that can eliminate the effects of uncertainty caused by discrete decision-making, such as that based on pain assessment scales^22–25^. However, only a few studies have actively employed continuous data, such as vital signs, in pain analysis. Adjei et al. predicted pain by quantifying pain sensitivity by analyzing the electrocardiogram (ECG) waveforms of 17 patients who underwent varicose vein surgery; however, the small number of patients and the use of questionnaires in pain assessment made it difficult to apply this method in unconscious patients^26^. There have also been attempts to detect pain through machine-learning analysis of electroencephalogram (EEG) and images^27–30^; however, they are difficult to interpret and cannot be used for continuous assessment. To the best of our knowledge, no studies have performed objective, continuous, and semi-automatic evaluation of pain in ICU patients. Therefore, this study aimed to investigate if AI analysis of vital signs could be used to automate the assessment of pain.

## Results

**Figure 1:**
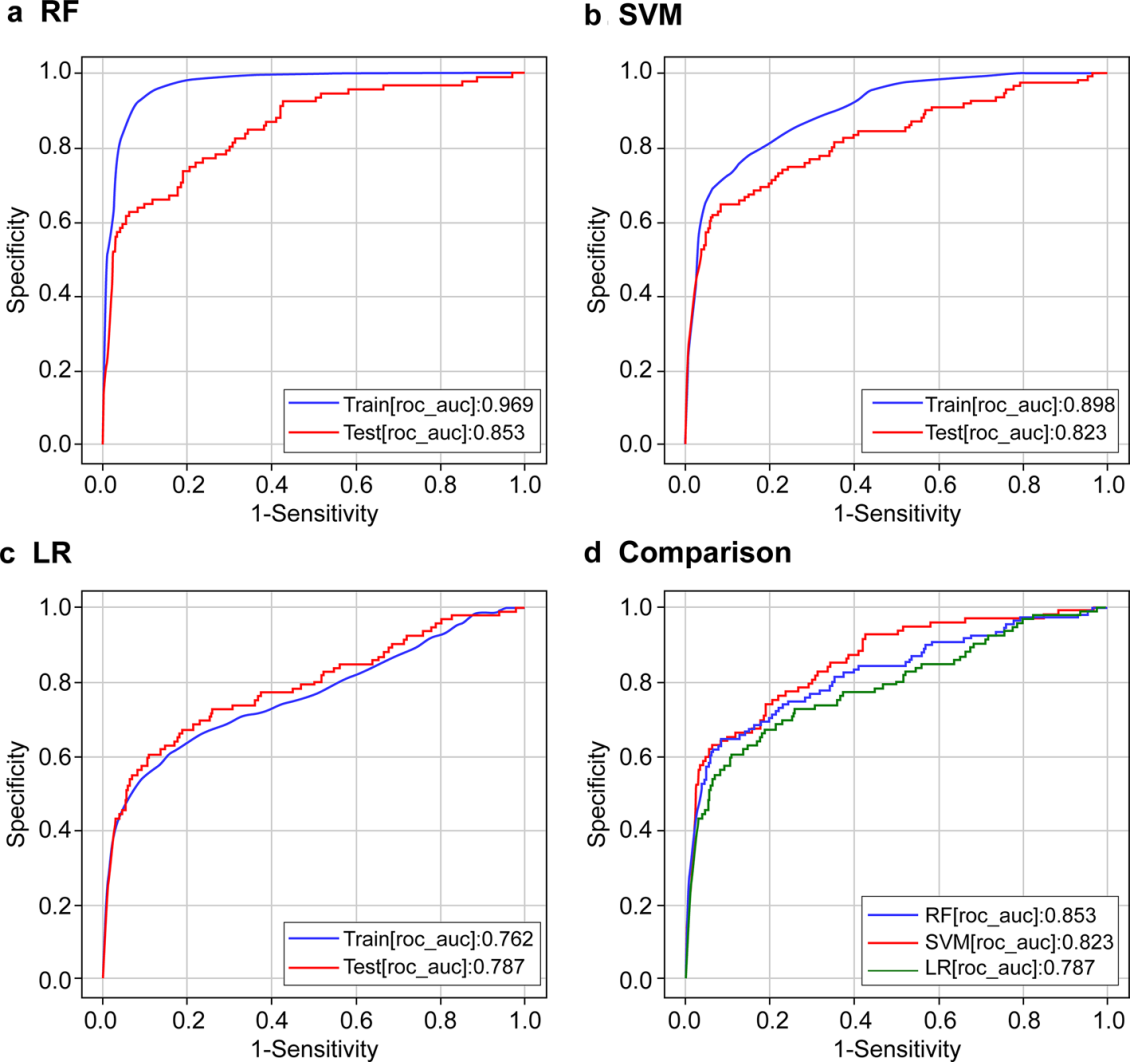
Prediction accuracy. (**a**–**c**) Accuracy of each machine-learning method. The blue line represents the accuracy in the training set, and the red line represents the accuracy in the test set. (**d**) Comparison of the verification data of the three machine-learning methods. The x-axis and y-axis represent the negative sensitivity and specificity in the ROC curve, respectively. The test accuracy depends on the extent to which the machine-learning model can correctly determine whether the CPOT score was < 2 or > 3. Accuracy is represented by AUROC; area of 1 represents the perfect test and that of 0.5 represents an inconclusive test.

In 11,527 eligible patients, CPOT assessment was performed 117,190 times and 3,925 patients (3.3%) were determined to experience pain with a score of ≥ 3. Although the level of sedation (Richmond Agitation-Sedation Scale; RASS) was assessed almost simultaneously, 42.8% of the patients were determined to be calm (0 points), 8.3% aggressive (≥ 1 point), and 48.9% sedated (≤ − 1 point). Furthermore, 21.7% of the ratings indicated delirium (see Supplementary Table S1 online). As mentioned in the Methods section, we used three machine-learning methods (RF, SVM, and LR) to predict the presence of pain at the time of CPOT evaluation using parameters, such as vital signs, age group, and sedation level. The hyperparameter that derived the highest accuracy in each model was selected via a grid search (SVM: C = 10, gamma = 10, kernel = rbf. RF: max_depth = 14, estimates = 17, criterion = gini, LR: C = 1.0, tol = 0.0001). The results demonstrated that RF provided the highest area under the receiver operating characteristic curve (AUROC) (AUROC for test data: RF: 0.853; SVM: 0.823; LR: 0.787; Fig. 1). A review of the feature importance of the RF model showed that RASS exhibits a higher contribution rate as compared to the other predictors (Supplementary Table S3). Table 1 lists the results obtained by varying the sensitivity and specificity while using the RF model, which demonstrates the highest prediction accuracy. These results are based on the oversampling performed in the proposed method. By contrast, the results obtained using raw data without oversampling are presented in Fig. S2 and Table S4 (Supplementary Information).

**Table 1:** Sensitivity, specificity, and threshold values at different operating points for RF-based pain tracking; the AUROC value of the prediction model equaled 0.853.

Condition	Sensitivity	Specificity	Threshold
Sensitivity at predefined value	0.6	0.942	0.736
	0.7	0.809	0.752
	0.8	0.691	0.742
	0.9	0.576	0.703
Specificity at predefined value	0.868	0.6	0.718
	0.791	0.7	0.742
	0.736	0.8	0.768
	0.637	0.9	0.746
Optimal point	0.736	0.807	0.770

## Discussion

In this study, we examined if machine learning can be used for continuous and automatic pain assessment and to obtain results similar to those of traditional CPOT assessments performed by nurses using only vital signs and other data. We found that the pain experienced by ICU patients can be assessed objectively, continuously, and semi-automatically using machine learning with an accuracy of up to AUROC 0.853. In our experiments, RF demonstrated the best prediction performance. Our study is novel because we demonstrated the usefulness of vital signs that are measured continuously and constantly in the ICU and were previously considered ineffective in the evaluation of pain.

Severely ill adult patients experience moderate-to-severe pain at rest and during procedures of standard care^1,31^; intense pain can have adverse effects, such as immunosuppression and cardiac and respiratory failure^32^. Currently, an objective index, such as CPOT, is used when an ICU patient cannot express pain; however, continuous evaluation is difficult because of the patient’s condition. Particularly, during the night or when the staff is busy, pain evaluation and care tend to be delayed. A consistent approach is important for pain assessment and management^33^, especially regarding the use of opioids, the main analgesics in ICUs, which require careful dose adjustments to balance the benefits and potential risks^34–38^. To achieve this goal, continuous evaluation of pain is crucial; however, pain evaluation using the conventional methods only corresponds to a specific time point. The results of this study suggest that RASS, an index of sedation, is the only parameter among all the variables that need to be evaluated each time and that pain evaluation can be semi-automated using vital signs.

Recent research using machine learning and AI has corroborated the usefulness of analyzing data that are recorded continuously over time, such as vital signs, to gain better insights. In line with the present study, a previous machine-learning model analyzed the electronic medical record texts of 2695 patients with breast cancer undergoing chemotherapy and automatically extracted pain and other information with AUROC of 0.82^39^. Furthermore, a median accuracy of 70% was achieved when a deep-learning model—based on a combination of convolutional and recurrent neural networks—that automatically tracks both levels of consciousness and delirium using frontal EEG signals was employed, which allowed the latter model to predict the differences in RASS levels to within 1 point^40^. In a previous study, we continuously analyzed blood pressure values in patients with septic shock and found that both the extent of decrease in blood pressure and the duration of blood pressure affect the prognosis^41^. The use of text and EEGs in medical records may improve the accuracy of pain prediction; however, they cannot always be measured.

In conclusion, pain can be objectively, continuously, and semi-automatically predicted using machine learning. Of the methods tested, the prediction accuracy was the highest with the RF model. Regarding the limitations of our research and future challenges, it is necessary to improve the prediction accuracy in diverse situations. In this study, we were also able to predict pain with a high degree of accuracy, although not 100%. Clinical limitations in this regard include time differences in pain assessment and recording, and heterogeneity that occurred as a result of the assessments being performed by humans. To address these issues, we plan to develop a bedside device using the prediction algorithm proposed in this study. Subsequently, it is necessary to improve the prediction accuracy in other environments to verify the results. The use of an automated and continuous pain assessment algorithm may allow for continuous pain assessment in all situations. It has the potential to not only immediately help relieve the pain in patients who cannot communicate but also increase their life expectancy. Therefore, more studies are warranted in a wider variety of scenarios for further evidence.

## Methods

### Dataset

This retrospective observational study was performed in the ICU of Tohoku University Hospital, Sendai, Japan. Ethical approval was obtained from the Ethics Committee of the Tohoku University Graduate School of Medicine (reference number 2018–1–650). The requirement for informed consent was waived due to the retrospective design by the Ethics Committee of Tohoku University Graduate School of Medicine. All methods were performed in accordance with the relevant guidelines and regulations as per the Declaration of Helsinki. The patients’ data will be deleted as soon as the current paper is published. The study was registered in a database in advance (ID: R000047019 UMIN000041179, URL: https://www.umin.ac.jp/ctr/index.htm). Patients who met the following inclusion criteria were enrolled at ICU admission between October 2016 and October 2019: (1) age ≥ 20 years; (2) CPOT, RASS, and confusion assessment method for the intensive care unit (CAM-ICU) were evaluated at least five times per patient; and (3) ECG and arterial pressure were monitored for ≥ 30 min before employing CAM-ICU. Data of the following patients whose vital signs differed significantly from those of the general adult population were excluded: (1) pregnant patients; (2) cardiopulmonary bypass recipients; and (3) those with organ transplants (i.e. organ recipients), ventricular assist devices, extracorporeal membrane oxygenation, intra-aortic balloon pumps, or do-not-resuscitate orders. The final dataset included 11,527 patients. The commonest analgesic and sedative were fentanyl and dexmedetomidine. All data were retrieved from the institution’s electronic medical record system (PrimeGaia, Nihon Kohden Corporation, Tokyo, Japan). No patient-identifying information was recorded.

### CPOT, RASS, and CAM-ICU

To assess the pain levels of all eligible patients, we used CPOT^11^ as the target to train the model. CPOT assessment was performed by ICU nurses every 8 h and when obvious pain was observed. CPOT includes nine levels (0–8). RASS^42^ was used to assess the sedation level. Delirium was assessed using CAM-ICU^43^. Furthermore, RASS and CAM-ICU assessments were performed concurrently with CPOT assessment. CPOT, RASS, and CAM-ICU evaluations were performed by multiple nurses to ensure agreement between them. If there was a difference in opinion, the intensivist made the final decision and approved the evaluations.

### Analgesic management protocol

All patients were managed using a pain management protocol of the hospital (see Supplementary Fig. S1 online).

### Vital signs

Heart rate, pulse oximetry, arterial oxygen saturation, and arterial blood pressure were recorded every minute. Arterial pressure was continuously monitored in either the left or right radial artery. Respiratory rate was measured using electrocardiographic impedance. The noise corresponding to each variable was removed according to the method described in Supplementary Table S2.

### Machine learning

We used three machine-learning methods with different characteristics (Table 2) according to the report by Saberioon et al.^44^.

**Table 2:** Brief schema of the machine learning methods applied in this study.

	Support vector machine	Random forest	Logistic regression
Explanation	A method of constructing a two-class pattern classifier using a linear input element. This is one of the supervised-learning-based pattern recognition models	A method of constructing numerous decision trees and carrying out a majority vote. It requires a large amount of data but can make highly accurate predictions	Calculates the class membership probability for two categories by fitting the log odds and explanatory variables to a model
Speed	**Low** Since the number of calculations is equal to the square of the number of data, this method can be computationally intensive, and the computational speed can be significantly low	**Moderate** Depending on the number of data and the number of dimensions chosen to build a single tree, it is often faster than SVM when the data size is large	**High** It utilizes stochastic gradient descent, and once the gradient is obtained, it can quickly find the solution and can be easily applied to large datasets
Accuracy	**High for a simplified problem** Classifies the data by subtracting a separation hyper-plane, which is suitable for two classifications; however, for multiple classifications, it creates a separation hyper-plane, which renders it difficult to execute and less accurate	**High when the volume of data is large** When solving simple problems with a small number of data, the accuracy is low owing to unavailability of data required for the large number of calculations. In contrast, when the data size is large, the accuracy is high, and overlearning is unlikely to occur	**Low** Performs calculations by applying a sigmoidal function to the output of a multiple regression. If the target variable tends to be in a single direction, the probability of correct classification is high. However, if there is a skewed class bias in the feature space, classification may be difficult to perform
Calibration	**Difficult** Kernels, regularization penalties, slack variables, etc. need to be adjusted. It is also necessary to regularize and standardize the training data in advance	**Easy** Only the depth and number of decision trees need to be adjusted. Further, regularization and standardization of the training data are not required	**Easy** Only the adjustment of slack variables is necessary. Further, regularization and standardization of the training data are not required

The table presents a comparative representation of the three machine learning methods.

### Prediction model

**Figure 2:**
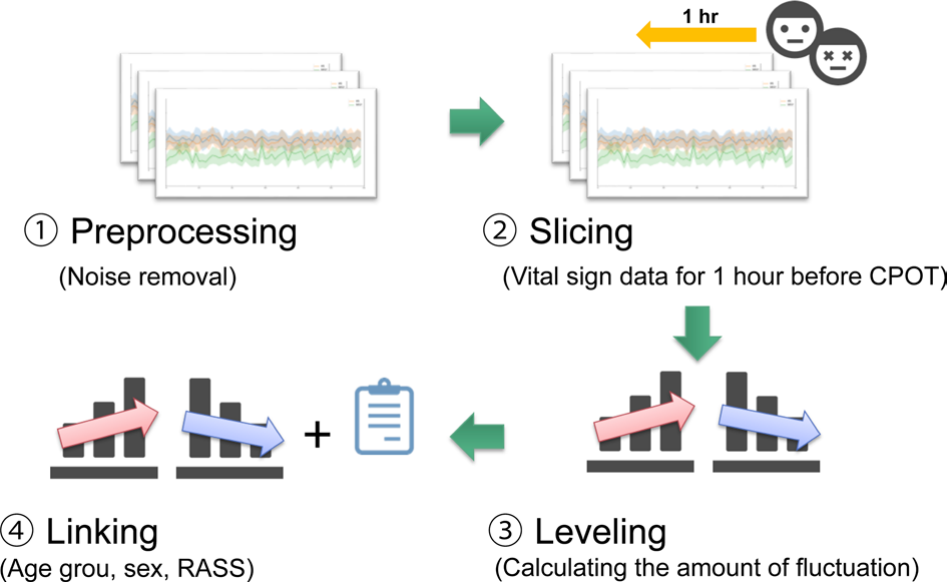
Procedure for creating training data.

Patient pain at the time of CPOT evaluation was predicted using machine learning and vital signs up to that time. The predictors were systolic and diastolic arterial blood pressure, pulse rate, and respiratory rate (one record/min) as vital signs as well as the patient’s sex, age group (20–44, 45–64, and 65 years), and RASS score in the last 3 h. Figure 2 illustrates the procedure used to create the training data. CAM-ICU was used for the exploratory analysis but not for the construction of the final model.

First, the noise was removed from the data of the vital signs according to the noise requirements in Supplementary Table S2 (preprocessing). Second, the vital signs from one hour before the CPOT evaluation were extracted (slicing). Third, the rising and falling fluctuations were calculated and integrated into the section in which 90% or more of the vital signs were present. To mitigate the influence of individual differences between patients, the integrated value of the amount of fluctuation from 1–2 h after entering the ICU was calculated every minute (60 records) and the fluctuations corresponding to each patient were calculated. The records were normalized based on the maximum and minimum values of the integrated value of the quantity (leveling), which provided a numerical value that indicated the magnitude of the variations in each patient. Finally, the age group, sex, and sedation score were assigned to the training data.

### Model evaluation

For predicting pain, CPOT values from 0 to 2 were considered negative and scores of ≥ 3 were considered positive. Since these data were unbalanced, oversampling was performed on the positive group. In terms of the oversampling method, Synthetic Minority Over-sampling Technique (SMOTE), Borderline SMOTE, and Adaptive Synthetic Sampling (ADASYN) were verified, and ADASYN, which showed the highest accuracy, was adopted. In machine learning, precision was compared between random forest (RF), support vector machine (SVM), and logistic regression (LR) models. To evaluate the generalization performance, the data were randomly classified between the training and validation data in a ratio of 9:1 and 10 cross-validations were performed. A grid search was performed on the training data to select the hyperparameter that derived the highest accuracy in each of the models. Furthermore, the harmonic means of the sensitivity, specificity, and AUROC were calculated.

### Statistical analysis

Data analysis was performed using JMP v15 (SAS Institute Inc., Cary, NC, USA). Normally distributed data were reported as mean ± standard deviation and non-normally distributed data as median and interquartile range. AUROC was used to compare the accuracy, which was evaluated as low (0.5–0.7), moderate (0.7–0.8), and high (≥ 0.8).

## Supplementary Information

Supplementary Information

## Data Availability

The datasets generated during and/or analyzed during the current study are available in the UMIN-ICDR repository at http://www.umin.ac.jp/icdr/index–j.html with permission from the authors.
