# Supplementary material for: Semi-automated tracking of pain in critical care patients using artificial intelligence: a retrospective observational study
Source: Sci Rep. 2021 Mar 4;11:5229. doi: 10.1038/s41598-021-84714-8 (PMC7933166; doi:10.1038/s41598-021-84714-8)
Supplement: Supplementary file 1 — Supplementary Information [file 41598_2021_84714_MOESM1_ESM.docx]

**Semi-automated tracking of pain in critical care patients using artificial intelligence: a retrospective observational study**

Naoya Kobayashi^1^*, Takuya Shiga^1^, Saori Ikumi^1^, Kazuki Watanabe^2^, Hitoshi Murakami^2^, Masanori Yamauchi^1^

^1^ Department of Anesthesiology and Perioperative Medicine, Tohoku University Graduate School of Medicine, Miyagi, Japan

^2^ Hitachi Solutions East Japan, Ltd., Sendai, Miyagi, Japan

*Corresponding author: Naoya Kobayashi

Department of Anesthesiology and Perioperative Medicine, Tohoku University Graduate School of Medicine, 2-1 Seiryo-machi, Aoba-ku, Sendai, Miyagi 980–8575, Japan

Tel: +81-22-717-7321

Fax: +81-22-717-7325

Email: dynamis_air@yahoo.co.jp

**Supplementary Information**

**Supplementary Table S1.** Patient characteristics

| Characteristics |  |
| --- | --- |
| Number of patients | 11,527 |
| Number of assessments ^a^ | 299,206 |
| CPOT: *N* |  |
| Total number of assessments | 117,190 |
| 0–2 | 113,265 |
| ≥ 3 | 3,925 |
| RASS: *N* |  |
| Total number of evaluations | 136,034 |
| 4 | 111 |
| 3 | 600 |
| 2 | 2,899 |
| 1 | 7,727 |
| 0 | 58,174 |
| –1 | 21,608 |
| –2 | 13,303 |
| –3 | 14,663 |
| –4 | 11,766 |
| –5 | 5,183 |
| CAM–ICU: *N* |  |
| Total number of evaluations | 71,497 |
| Negative (CPOT: 0–2) | 55,999 |
| Positive (CPOT: > 3) | 15,498 |
| Age: year, median (IQR) | 65 (73, 52) |
| Age group (years of age): *Number of evaluations* |  |
| 20-44 | 5,758 |
| 45-64 | 13,971 |
| 65- | 21,831 |
| Male: *N* (%) | 6,660 (58.0%) |
| Days in ICU: day, median (IQR) | 1 (3, 1) |
| Days in ICU: hour, median (IQR) | 21.6 (62.6, 17.6) |
| APACHE II at ICU admission: median (IQR) | 7 (17, 10) |
| ICU admission diagnosis: *N* (%) |  |
| Heart | 1,321 |
| Aorta and vessels | 1,208 |
| Kidney | 487 |
| Lung and mediastinum | 1,155 |
| Digestive organs | 3,154 |
| Metabolic and endocrine organs | 198 |
| Orthopedics | 305 |
| Genital organs | 1,053 |
| Obstetrics | 123 |
| Neurosurgery | 654 |
| Others | 1,869 |
| Average infusion rate: mg/h/kg (number of patients receiving each drug, %) | |
| Propofol | 6.05 (N: 1,802, 15.6%) |
| Hydromorphone | 3.36 (N: 31, 0.3%) |
| Dexmedetomidine | 7.06 (N: 2,389, 20.7%) |
| Fentanyl | 1.93 (N: 2,878, 25.0%) |
| Ketamine | 2.96 (N: 15, 0.1%) |
| Midazolam | 3.56 (N: 289, 2.5%) |

^a^ Number of times one or more evaluations (CPOT, RASS, or CAM-ICU) were performed

Abbreviations: CPOT, Critical-Care Pain Observation Tool; RASS, Richmond Agitation-Sedation Scale; CAM–ICU, confusion assessment method for the intensive care unit; ICU, intensive care unit; IQR, interquartile range

**Supplementary Table S2.** Noise cancelation for arterial blood pressure

| Factors | Requirements |
| --- | --- |
| Systolic pressure | < 35, 300 < |
|  | < 80 changes from previous record |
|  | < 40 changes are measured twice or more consecutively |
| Diastolic pressure | < 20 |
| Systolic and diastolic pressure | (Systolic pressure) - (Diastolic pressure) < 15 |
| Heart rate | < 0, 250 < |
| Respiratory rate | < 3, 81 < |

For records corresponding to one-minute intervals where even one of the above items applies, all vital signs were excluded from the analysis.

**Supplementary Table S3.** Feature importance of the random forest model

| Predictors | Importance |
| --- | --- |
| RASS (-5 to +4) | 0.426 |
| Age group (20-44, 45-64, and 65- years of age) | 0.013 |
| Gender (Male, female) | 0.030 |
| Diastolic arterial pressure + | 0.044 |
| Diastolic arterial pressure – | 0.041 |
| Systolic arterial pressure + | 0.043 |
| Systolic arterial pressure – | 0.044 |
| Mean arterial pressure + | 0.042 |
| Mean arterial pressure – | 0.044 |
| Pulse rate + | 0.045 |
| Pulse rate – | 0.046 |
| Heart rate + | 0.037 |
| Heart rate – | 0.038 |
| Respiratory rate + | 0.050 |
| Respiratory rate – | 0.054 |

The "+" and "–" after each parameter indicate the upward or downward variation from the previous value, respectively. For example, in the upward direction, a downward variation from the previous value is calculated as 0. RASS: Richmond Agitation-Sedation Scale.

**Supplementary Table S4.** Values of sensitivity, specificity, and threshold values at different operating points for RF-based pain tracking obtained using raw data without oversampling. The AUROC value of the prediction model is 0.798.

| Condition | Sensitivity | Specificity | Threshold |
| --- | --- | --- | --- |
| Sensitivity at predefined value | 0.6 | 0.900 | 0.736 |
|  | 0.7 | 0.831 | 0.752 |
|  | 0.8 | 0.547 | 0.742 |
|  | 0.9 | 0.528 | 0.714 |
| Specificity at predefined value | 0.758 | 0.6 | 0.718 |
|  | 0.747 | 0.7 | 0.742 |
|  | 0.714 | 0.8 | 0.768 |
|  | 0.582 | 0.9 | 0.746 |
| Optimal point | 0.703 | 0.865 | 0.776 |

**Supplementary Figure S1**. CPOT was assessed by intensive care nurses every 8 hours and when obvious pain was observed. NRS: numerical rating scale; CPOT: Critical-Care Pain Observation Tool; PCEA: patient-controlled epidural analgesia.

**Supplementary Figure S2**. Prediction accuracy trends without oversampling. **a-c**: Accuracy of each machine-learning method. The blue line represents the accuracy in the training set, and the red line represents the accuracy in the test set. **d**: Comparison of the verification data of the three machine-learning methods. The x-axis and y-axis represent the negative sensitivity and specificity in the ROC curve, respectively. The test accuracy depends on the extent to which the machine-learning model can correctly determine whether the CPOT score was < 2 or > 3. Accuracy is represented by AUROC; area of 1 represents the perfect test and that of 0.5 represents an inconclusive test.
